# Supplementary material for: Prognostic Significance of Cardiac Magnetic Resonance in Left Atrial and Biventricular Strain Analysis during the Follow-Up of Suspected Myocarditis
Source: J Clin Med. 2023 Jan 6;12(2):457. doi: 10.3390/jcm12020457 (PMC9862408; doi:10.3390/jcm12020457)
Supplement: Supplementary file 1 [file jcm-12-00457-s001.zip › jcm-2042128-supplementary.pdf]

Detailed scanning parameters of 3 vendors

Machine 1(Philips): 32-channel cardiac surface phased-array coil, parallel imaging technique was used, PE=2. FOV was adapted to the patient size. Mean RR was about 0.6-1.0s depending on the heart rate.

T2WI-STIR images: black blood T2-weighted imaging short Tau inversion recovery (T2 STIR) sequence. TR=1250, TE=75, matrix=224 mm×228 mm, flip angle=45°, slice thickness=8-10 mm, gap=0 mm.

Cine imges: Balanced steady-state free precession(bSSFP) sequence. TR=2.9, TE=1.45, matrix=152 mm×150 mm, flip angle=45°, slice thickness=8-10 mm, gap=0 mm, bandwith=2284.1 Hz/pixel, number of heart beat was 5-10 per apnea depending on the heart rate. 30 phases per cardiac cycle. 25 views per segment.

LGE image: Phase-sensitive inversion recovery sequence(PSIR) sequence. TR=6.1, TE=3.0, matrix=220 mm×160 mm, flip angle=25°, slice thickness=8-10 mm, gap=0 mm, bandwith=228.2 Hz/pixel, number of heart beat was 6-12 per apnea depending on the heart rate. 30 views per segment.

Machine 2(Siemens): 32-channel cardiac surface phased-array coil, parallel imaging technique was used, PE=2. FOV was adapted to the patient size. Mean RR was about 0.6-1.0s depending on the heart rate.

T2WI-STIR images: black blood T2-weighted imaging short Tau inversion recovery (T2 STIR) sequence. TR=800, TE=72, matrix=256 mm×256 mm, flip angle=45°, slice thickness=8-10 mm, gap=0 mm.

Cine images: True fast imaging with steady-state precession sequence. TR=2.7, TE=1.35, matrix=256 mm×256 mm, flip angle=50°, slice thickness=8-10 mm, gap=0 mm, bandwith=814 Hz/pixel, 25 phases per cardiac cycle, number of heart beat was 5-10 per apnea depending on the heart rate. 30 views per segment.

LGE image: Phase-sensitive inversion recovery sequence(PSIR) sequence. TR=5.4, TE=2.7, matrix=370 mm×350 mm, flip angle=20°, slice thickness=8-10 mm, gap=0 mm, bandwith=465 Hz/pixel, number of heart beat was 6-12 per apnea depending on the heart rate. 35 views per segment.

Machine 3(GE Healthcare): 32-channel cardiac surface phased-array coil, parallel imaging technique was used, PE=2. FOV was adapted to the patient size. Mean RR was about 0.6-1.0s depending on the heart rate.

T2WI-STIR images: black blood T2-weighted imaging short Tau inversion recovery (T2 STIR) sequence. TR=1690, TE=68, matrix=160 mm×192 mm, flip angle=45°, slice thickness=8-10 mm, gap=0 mm.

Cine images: Fast imaging employing steady state acquisition sequence. TR=3.6, TE=1.8, matrix=256 mm×192 mm, flip angle=60°, slice thickness=8-10 mm, gap=0 mm, bandwith=100 Hz/pixel, 25 phases per cardiac cycle, number of heart beat was 5-10 per apnea depending on the heart rate. 20-24 views per segment.

LGE images: Phase-sensitive inversion recovery sequence(PSIR) sequence. TR=5.3, TE=2.5, matrix=220 mm×192 mm, flip angle=35°, slice thickness=8-10 mm, gap=0 mm, bandwith=41.67 Hz/pixel, number of heart beat was 6-12 per apnea

depending on the heart rate. 24 views per segment.

Table S1. Intra-observer and inter-observer variability of different strain parameters

Parameter	Variability	ICC	95% CI
LVGRS	Intra-observer	0.982	0.947-0.994
	Inter-observer	0.944	0.834-0.982
LVGCS	Intra-observer	0.980	0.940-0.993
	Inter-observer	0.958	0.875-0.986
LVGLS	Intra-observer	0.984	0.952-0.995
	Inter-observer	0.966	0.900-0.989
RVGRS	Intra-observer	0.974	0.922-0.991
	Inter-observer	0.946	0.843-0.922
RVGCS	Intra-observer	0.979	0.935-0.993
	Inter-observer	0.922	0.782-0.974
RVGLS	Intra-observer	0.987	0.960-0.996
	Inter-observer	0.972	0.916-0.991
LAƐs	Intra-observer	0.984	0.952-0.995
	Inter-observer	0.968	0.905-0.990
LAƐa	Intra-observer	0.898	0.718-0.966
	Inter-observer	0.818	0.539-0.937
LAƐe	Intra-observer	0.977	0.931-0.993

	Inter-observer	0.936	0.818-0.979
LASRs	Intra-observer	0.903	0.727-0.968
	Inter-observer	0.815	0.523-0.936
LASRe	Intra-observer	0.964	0.896-0.988
	Inter-observer	0.950	0.856-0.984
LASRa	Intra-observer	0.827	0.558-0.940
	Inter-observer	0.757	0.416-0.914

Table S2. Univariate association of the second laboratory and CMR parameters with MACE

Parameter	HR (95% CI)	P value
Peak level CK (U/L)	0.999 (0.995, 1.002)	0.470
Peak level hs-TNI(ng/mL)	0.961 (0.828, 1.115)	0.601
Peak level NT-proBNP(pg/mL)	1.000 (1.000, 1.001)	0.322
Peak level CRP(mg/L)	1.018 (0.959, 1.080)	0.555
WBC count(*10^9/L)	1.030 (0.894, 1.187)	0.682
Positive T2WI	0.898 (0.256, 3.155)	0.867
Positive LGE	0.680 (0.236, 1.964)	0.477
Positive “LLC”	0.042 (0.000, 41.633)	0.367
LVEF, %	0.961 (0.909, 1.017)	0.168
RVEF, %	0.979 (0.944, 1.016)	0.267

LAEF, %	0.975 (0.937, 1.014)	0.210
LAPEF, %	1.008 (0.958, 1.061)	0.748
LAAEF, %	1.012 (0.980, 1.045)	0.475
LVGRS, %	0.986 (0.937, 1.038)	0.593
LVGCS, %	1.092 (0.939, 1.271)	0.253
LVGLS, %	0.991 (0.873, 1.125)	0.888
RVGRS, %	1.012 (0.987, 1.038)	0.334
RVGCS, %	0.983 (0.934, 1.035)	0.521
RVGLS, %	1.020 (0.975, 1.067)	0.400
Ɛs, %	1.000 (0.957, 1.046)	0.984
Ɛa, %	1.032 (0.955, 1.115)	0.431
Ɛe, %	0.984 (0.925, 1.046)	0.603
SRs, s^{-1}	0.818 (0.370, 1.809)	0.620
SRe, s^{-1}	1.148 (0.682, 1.933)	0.604
SRa, s^{-1}	0.986 (0.535, 1.818)	0.964
